# Development of PHITS graphical user interface for simulation of positron emitting radioisotopes production in common biological materials during proton therapy

**DOI:** 10.1093/jrr/rrac010

**Published:** 2022-03-28

**Authors:** Mehrdad Shahmohammadi Beni, Kwan Ngok Yu, M Rafiqul Islam, Hiroshi Watabe

**Affiliations:** Department of Physics, City University of Hong Kong, Tat Chee Avenue, Kowloon Tong, Hong Kong; Division of Radiation Protection and Safety Control, Cyclotron and Radioisotope Center, Tohoku University, 6-3 Aoba, Aramaki, Aoba-ku, Sendai, Miyagi 980-8578, Japan; Department of Physics, City University of Hong Kong, Tat Chee Avenue, Kowloon Tong, Hong Kong; Graduate School of Biomedical Engineering, Tohoku University, Sendai 980-8579, Japan; Graduate School of Biomedical Engineering, Tohoku University, Sendai 980-8579, Japan; Division of Radiation Protection and Safety Control, Cyclotron and Radioisotope Center, Tohoku University, 6-3 Aoba, Aramaki, Aoba-ku, Sendai, Miyagi 980-8578, Japan

**Keywords:** Monte Carlo (MC), nuclear radiation, Particle and Heavy Ion Transport Code System (PHITS), positron, proton therapy

## Abstract

The Monte Carlo (MC) method is a powerful tool for modeling nuclear radiation interaction with matter. A variety of MC software packages has been developed, especially for applications in radiation therapy. Most widely used MC packages require users to write their own input scripts for their systems, which can be a time consuming and error prone process and requires extensive user experience. In the present work, we have developed a graphical user interface (GUI) bundled with a custom-made 3D OpenGL visualizer for PHITS MC package. The current version focuses on modeling proton induced positron emitting radioisotopes, which in turn can be used for verification of proton ranges in proton therapy. The developed GUI program does not require extensive user experience. The present open-source program is distributed under GPLv3 license that allows users to freely download, modify, recompile and redistribute the program.

## INTRODUCTION

Monte Carlo (MC) simulation is an established tool which is extensively employed in simulation of nuclear radiation transport in matter, thanks to that the stochasticity of radiation interaction with matter can be conveniently considered in MC models [1–11]. The MC method is widely used in modeling radiation therapy and nuclear imaging. A variety of MC packages were developed for radiation transport and dosimetry, some of the well-known and widely used examples being; Monte Carlo N-Particle (MCNP) (https://mcnp.lanl.gov/), Geant4 (https://geant4.web.cern.ch/node/1), FLUKA (http://www.fluka.org/fluka.php), Particle and Heavy Ion Transport Code System (PHITS) (https://phits.jaea.go.jp/) and many others. These widely used MC packages have their own specific scripting syntaxes and format for setting up the simulation of radiation transport, such as geometry, material, source and tally definitions. Preparing the input scripts for these widely used MC packages are actually time consuming and error-prone processes and require user experience.

Interest in proton therapy has increased in recent years. In proton therapy, high energy protons are used to deliver doses to tumors. As a result of the interaction between high energy protons with tissues, the tissue elements would be activated [12]. ^1^H, ^12^C and ^16^O are the commonest elements found in body tissues, with ^12^C and ^16^O being able to produce ^11^C and ^15^O/^13^N, respectively, which are positron emitting radioisotopes and in turn generate annihilation photons within the patient’s body. It is remarked that ^1^H does not produce a stable positron emitter and will not be considered in most investigations.

Previously, Cho *et al*. [12] proposed to use the generated ^13^N positron emitters for proton range verification due to its low energy threshold that peaks at around 12 MeV. In their previous study, water-like gel and tissue-like gel phantoms were irradiated and scanned using an in-room positron emission tomography (PET) system. The proposed method was interesting and promising in that it could be used to enhance the effectiveness and accuracy of proton therapy. We refer interested readers to Cho *et al.* [12] for more information on this approach. Recently, Furuta and Sato [3] extensively discussed the medical applications of the Particle and Heavy Ion Transport Code System (PHITS) MC package and its features that would be useful in this particular field. In addition, the PHITS MC package was employed for estimating radiation doses in medical applications [13, 14]. Considering the wide applications of the PHITS MC package, we aimed to develop an open-source graphical user interface (GUI) program for the package that could be used to simulate a phantom composed of most commonly used materials during proton irradiations. A dedicated 3D visualizer based on OpenGL was also developed and bundled with the GUI program. The present work would be useful for determining the production of positron emitting radioisotopes (particularly ^13^N) for verification of proton ranges without requiring extensive user experience in MC method and code scripting. The present open-source program is distributed under GPLv3 license that allows users to freely modify, recompile and redistribute.

## MATERIALS AND METHODS

**Fig. 1.**
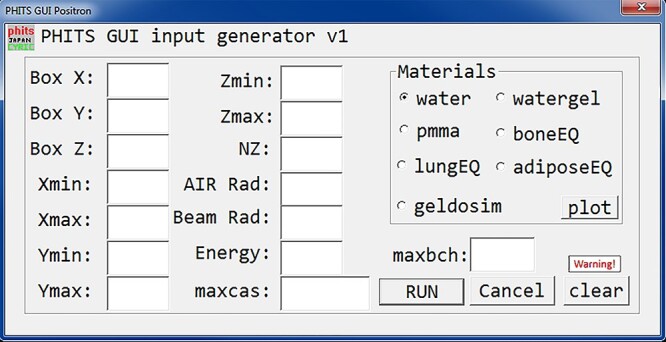
PHITS input generator GUI for proton irradiation of commonly used biological materials.

The present program can be divided into six main parts: (i) the main program, (ii) GUI control callback, (iii) GUI callback function, (iv) PHITS script generator, (v) linear interpolation and (vi) proton range subroutines. The main program and all the subroutines were written in the FORTRAN90 programming language. The OpenGL 3D plotter program was written in the C++ programming language that read the output of the main GUI program to plot the 3D structure. The developed GUI program is shown in Fig. 1.

**Fig. 2.**
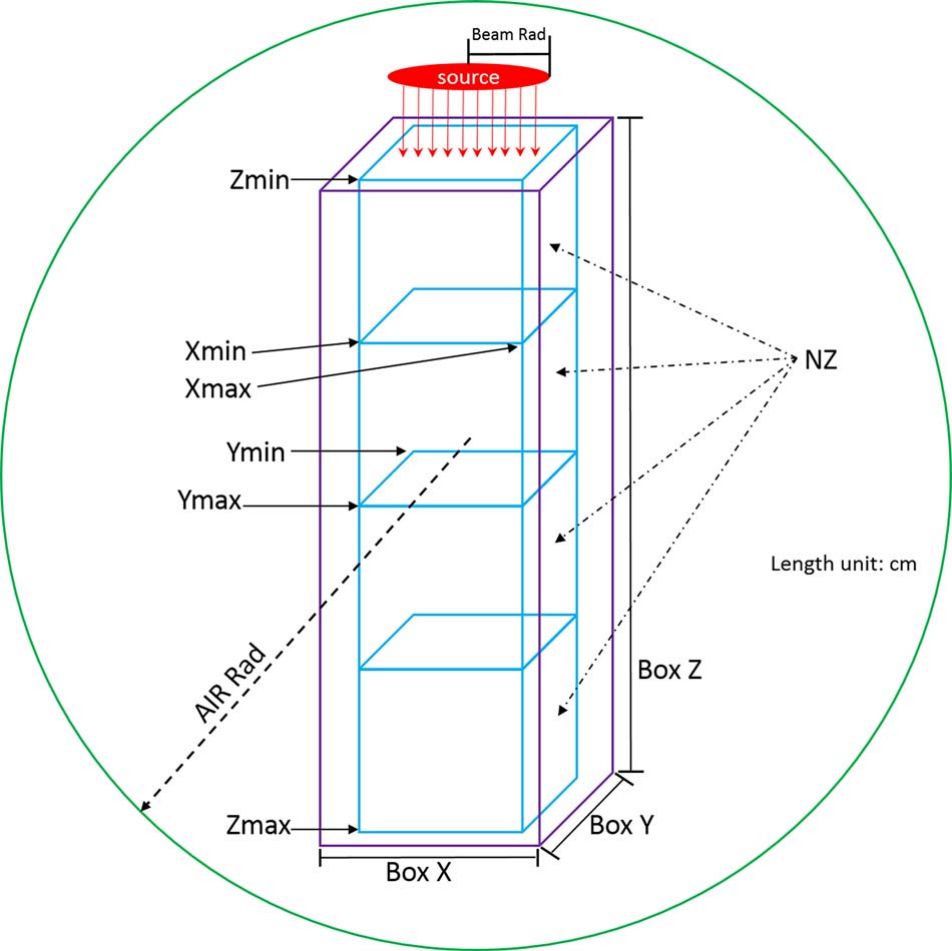
Schematic diagram showing each parameter used in the present GUI.

**Table 1:** Material composition and density of commonly used biological materials in proton therapy

Material	Composition (weight %)	Density (g/cm^3^)	Ref.
Water	H-11.1, O-88.9	1.00	[15]
Water gel	H-11.03, C-1.36, O-87.6	1.01	[16]
PMMA	H-8.05, C-59.99, O-31.96	1.18	[17]
Bone equivalent tissue	H-3.41, O-36.50, C-31.41, Ca-26.81, N-1.84, Cl-0.04	1.819	[17]
Lung equivalent tissue	H-8.46, O-18.14, C-59.38, Mg-11.19, N-1.96, Cl-0.1, Si-0.78	0.30	[18]
Adipose equivalent tissue	H-11.4, O-27.9, C-59.8, Na-0.1, N-0.7, Cl-0.1	0.94	[17]
Gel dosimeter	H-10.01, O-73.1, C-14.41, N-2.48	1.08	[19]

**Fig. 3.**
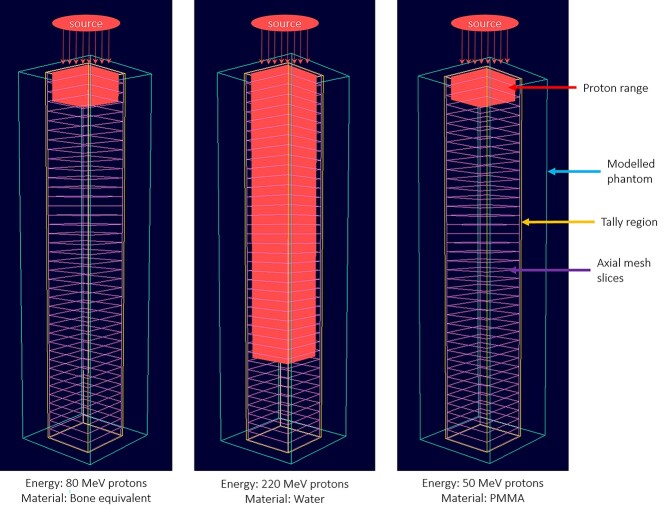
Snapshots from the OpenGL 3D plotter with region labeling. The phantom is irradiated from the top.

The present program simulated a rectangular parallelepiped with user defined dimensions, *Box X, Box Y* and *Box Z* (all in cm), which were placed in an air sphere centered at the origin (i.e. 0,0,0) with the user defined radius *Air Rad* (in cm). The variables *Xmin*, *Xmax*, *Ymin*, *Ymax*, *Zmin* and *Zmax* (all in cm) represented the tally region within the modeled rectangular parallelepiped. The variable *NZ* (integer number) defined the number of mesh slices along the z-axis (i.e. axially along the proton beam) at which the beam of protons is launched with a source radius defined by the *Beam Rad* (in cm) variable. It is possible for the users to change the beam size by defining Beam Rad, which is essentially the proton beam radius. The modeled geometry and tally region are shown schematically in Fig. 2. The energy of incident protons was defined using Energy (in MeV). In the present version of the developed GUI program, the beam has been fixed to a monoenergetic proton disk source, however, users can change the beam radius and energy. Users can change source parameters such as particle type and direction manually in the generated PHITS script using the GUI program. The number of histories and batch number were defined using *maxcas* (integer number) and *maxbch* (integer number), respectively. The users could choose seven different materials: (i) water [15], (ii) water gel [16], (iii) PMMA (acrylic) [17], (iv) bone equivalent [17], (v) lung equivalent [18], (vi) adipose equivalent [17], and (vii) gel dosimeter [19]. The present GUI program has the ability to model homogenous phantoms with one seamless material definition for each simulation scenario. The main goal of this version of GUI program was to model homogenous phantoms that were being widely used in proton irradiation experiments, such as those for proton range verification prior to proton therapy [20]. The material composition, density and relevant references of these materials are shown in Table 1.

**Fig. 4.**
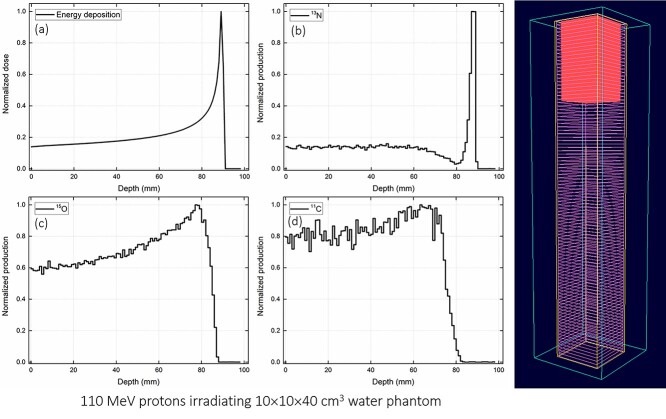
Test case 1: irradiation of water phantom with 110 MeV protons with (a) total energy deposition, distribution of (b) ^13^N, (c) ^15^O and (d) ^11^C production. The 3D plot has been obtained from the OpenGL plotter program.

**Fig. 5.**
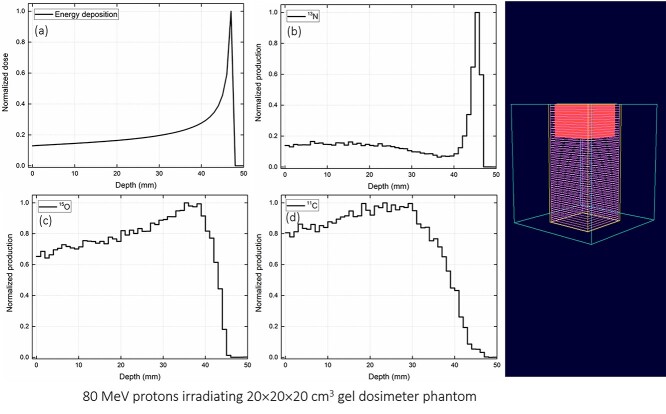
Test case 2: irradiation of gel dosimeter phantom with 80 MeV protons with (a) total energy deposition, distribution of (b) ^13^N, (c) ^15^O and (d) ^11^C production. The 3D plot has been obtained from the OpenGL plotter program.

**Fig. 6.**
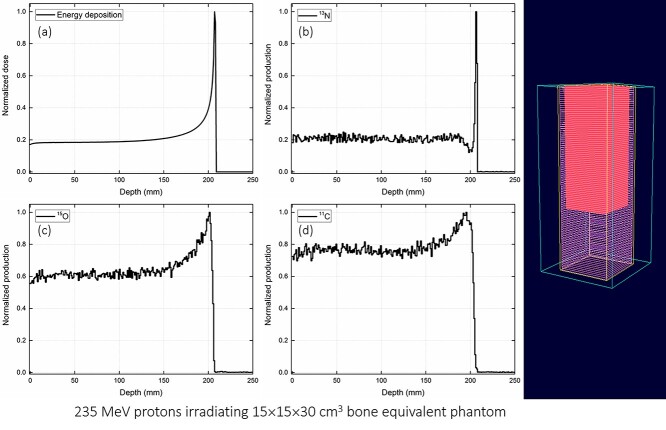
Test case 3: irradiation of bone equivalent phantom with 235 MeV protons showing (a) total energy deposition, distribution of (b) ^13^N, (c) ^15^O and (d) ^11^C production. The 3D plot has been obtained from the OpenGL plotter program.

After the user entered the inputs through the GUI, the program read all entries as characters, which were then converted into either real or integer numbers depending on the specific variable. The parameter limits are based on the user inputs for each variable that will be read by the GUI program in 256 characters string and then converted to single precision 4-byte real numbers which has seven digits of accuracy and a magnitude range from 10^−38^ to 10^38^. Another limitation would be the format of the numbers in Fortran. The core of the present GUI program that generates the entire PHITS script requires number formatting for writing the PHITS script. The current format of the GUI variables enables the users to input geometry sizes up to four digits and less than 2000 cm in length and tally region up to three digits (i.e. 999 cm); these can be easily increased depending on the user demand by changing the format length and recompiling the program. The control callback subroutine handled the asynchronous callback when the corresponding button was pressed. The callback subroutine read all the user inputs from the GUI, converted characters to either real or integer numbers and called the PHITS script generator subroutine. The PHITS script generator subroutine handled the generation of PHITS input code with specific formatting and syntaxes built into the PHITS script generator. The generated PHITS script from this subroutine was saved into an input file and the callback subroutine initiated the PHITS MC package through a system call with the generated input code. It is remarked that for automatic PHITS MC run, users would need to have PHITS installed on their computers.

The OpenGL 3D plotter program read the output from the GUI program. The linear interpolation and proton range subroutine were called from the callback function subroutine upon plotting. The interesting feature here was the visualization of proton ranges in 3D Cartesian coordinates, which was displayed along with the modeled phantom, tally region and axial mesh slices. The GUI program wrote the dimensions of the modeled phantom, tally region and number of axial mesh slices into output files. In addition, data on proton ranges for the seven different materials shown in Fig. 1 were obtained from SRIM (http://www.srim.org/) for incident proton energies ranging from 20 to 250 MeV. All the proton range data obtained from SRIM that were implemented into the program can be found on: https://figshare.com/articles/software/Development_of_PHITS_graphical_user_interface_for_simulation_of_positron_emitting_radioisotopes_production_in_common_biological_materials/17054864. These data were implemented into the proton range subroutine and interpolated (upon calling linear interpolation subroutine) to the nearest user defined incident proton energy. Using the obtained proton range, another output file would be generated containing the maximum projected range which would then be used by the OpenGL plotter program. Snapshots from the OpenGL 3D plotter program are shown in Fig. 3. In addition, mouse and keyboard support were implemented into the OpenGL plotter program. The users could use the mouse to rotate and move the object and zoom in and out with the keyboard arrow keys.

In the present GUI program, a loop function was implemented that prevented overwriting of the output code and results generated by the PHITS MC package; this would be particularly useful when users aimed to simulate a large number of cases/scenarios. The generated PHITS input script was numerically numbered and PHITS MC package outputs would be named with the user defined energy and material for ease of recognition and post processing. The present GUI program generates the entire PHITS script for proton irradiation of homogenous phantoms composed of seven different materials: (i) water, (ii) water gel, (iii) PMMA (acrylic), (iv) bone equivalent, (v) lung equivalent, (vi) adipose equivalent, and (vii) gel dosimeter. The user can intuitively change the parameters and immoderately run PHITS and see the output results. The present GUI program has the ability to execute PHITS by clicking a button and the users can immediately view the output results. The program generates all tally cards such as; title, parameters, source, material, surface and tallies automatically. The command for geometries that are RPP and SO for rectangular parallelepiped and air sphere centered at origin, respectively, will be printed in the PHITS script automatically. The user defined rectangular parallelepiped dimensions, (i.e. *Box X, Box Y, Box Z*) and air sphere radius (i.e. *Air Rad*) will be filled into RPP and SO in the surface card of the PHITS script by the program. The source card with proton projectile will also be automatically generated by the program and the user input beam radius (*Beam Rad*) and incident energy (i.e. *Energy*) will fill the source card accordingly. The product tally card with all its commands will also be generated by the program and the variables *Xmin, Xmax, Ymin, Ymax, Zmin, Zmax* and *NZ* will fill the tally card. The tally card option for generating output plots in the eps image format will be turned on automatically by the program so that users can easily view the simulation output results. The GUI program and all its source code can be found at: https://figshare.com/articles/software/Development_of_PHITS_graphical_user_interface_for_simulation_of_positron_emitting_radioisotopes_production_in_common_biological_materials/17054864.

Three different test cases were considered to validate the present GUI program: (i) 110 MeV protons irradiating 10 × 10 × 40 cm^3^ water phantom, (ii) 80 MeV protons irradiating 20 × 20 × 20 cm^3^ gel dosimeter phantom, and (iii) 235 MeV protons irradiating 15 × 15 × 30 cm^3^ bone equivalent phantom. The normalized dose and production of positron emitting radioisotopes versus depth were determined for every test case. In addition, the 3D plot was generated using the OpenGL 3D plotter program.

## RESULTS AND DISCUSSION

The test results from the present GUI program for three different cases have been shown in Figs 4–6. The input script was generated by the PHITS script generator subroutine, which was then automatically passed as an argument to the PHITS MC package. In the present study, the PHITS MC package version 3.1 for Microsoft windows was used. The PHITS MC package was compiled with the intel Fortran compiler (i.e. ifort compiler). The cross sectional data from ATIMA package was used to explain the interaction of proton with matter. The average statistical uncertainty (i.e. relative error) associated with the MC computations was found to be lower than 5%. Furthermore, no additional conversion coefficients were applied in the analysis of the output results. The GUI program execution takes less than a few seconds of CPU time and the PHITS execution highly depends on the number of histories set by the user. These were tested on a Microsoft Windows machine with dual Xeon E5-2630 v3 2.40 GHz CPUs.

The main difference of the present GUI program is that it generates the entire PHITS script and it is not limited to geometries and materials definition; this makes the present program easy and simple even for novice users. The user can intuitively change the parameters and immoderately run PHITS MC package and see the output results. This feature is especially good for pedagogical applications and medical staffs in clinical studies. Some notable tools such as SimpleGeo (http://theis.web.cern.ch/simplegeo/) is an interactive solid modeler that would help users design complex geometries for FLUKA, MCNP and PHITS MC packages, PHITS Interactive Geometry viewer in 3D (PHIG-3D) (bundled with PHITS MC package) that is commonly used for visualization of 3D geometries by reading PHITS input file; while this tool can enlarge, reduce and rotate geometries prepared in the PHITS input script. Another notable feature for defining geometrical features would be SuperMC (https://github.com/chunshen1987/superMC) that enables the conversion of a CAD file to the PHITS input format. These tools have higher flexibility; however, it would be rather difficult to be operated by beginners. There are also a variety of GUI programs developed for other MC packages such as Vised (http://www.mcnpvised.com/visualeditor/visualeditor.html) that was developed for the MCNP MC package, which enables visualization of the MCNP input script, geometrical debugging and visualization of particle tracks in a variety of applications [4, 21]. Vised requires MCNP input script to be loaded into the software which would be rather tedious for new users. In addition, operating Vised requires experience since incorrect inputs can lead to program crashes. Another notable GUI would be Wisp (http://geant4-resources.com/Geant4GUI/G4GUI.html) that is developed for Geant4 MC package. It has the ability to determine particle tracks, mean free path and dose deposition in almost any element from the periodic table, however, common biological materials that are of most interest in medical applications were not considered. In addition, Wisp can only determine dose and mean free path, while the production of radionuclides as a result of irradiation was not considered. The EGS Windows (https://rcwww.kek.jp/research/egs/user.html) is another general-purpose 3D viewer for the use EGS5 MC package; this tool is only for visualization of geometries and does not offer input script generator. Unlike other tools that would only assist users to generate 3D geometries, the present GUI program generates the full PHITS script and assigns numerical values to each command based on user inputs. Users do not need to install the present GUI program (as it is a portable executable software) or interact with any scripts or codes.

The results shown in Figs 4–6 were obtained from the PHITS MC package and then normalized. These results revealed that the energy deposition (i.e. dose) increases with the axial depth within the phantom (see Figs 4a, 5a and 6a), mainly as a result of energy loss of protons in the simulated media that leads to an increased stopping power [8]. The Bragg peak formation can be clearly seen in the energy deposition graphs, i.e. Figs 4a, 5a and 6a. Production of positron emitting radioisotopes such as ^13^N, ^15^O and ^11^C are also shown in Figs 4–6. These radioisotopes were generated as a result of proton interaction with the phantom material. In particular, the ^16^O(p,2p2n)^13^N, ^16^O(p,pn)^15^O and ^12^C(p,pn)^11^C nuclear reactions produced ^13^N, ^15^O and ^11^C radioisotopes, respectively. The present GUI program automatically generated the tally card for scoring the production of these positron emitting radioisotopes. This was indeed useful for users as it reduced the time for preparing the input script and prevented any potential errors in tally definition. As reported previously [12], the ^13^N peak was close to the actual Bragg peak and therefore it could be used for verification and monitoring of proton range during radiation therapy. This would be particularly useful for benchmarking the experimentally obtained results from PET systems using the MC method.

Snapshots from the developed OpenGL 3D plotter program is shown in Figs 4–6. The plotted geometry provides information about the modeled phantom, tally region, axial mesh slices and the proton range within the simulated phantom. The OpenGL plotter program would be useful for quick visualization of the modeled system and proton range in the phantom.

## CONCLUSIONS

In the present work, an open-source GUI program was developed for the PHITS MC package for proton irradiation of a homogeneous phantom made of most commonly used biological materials. The users could choose seven different materials: (i) water, (ii) water gel, (iii) PMMA, (iv) bone equivalent, (v) lung equivalent, (vi) adipose equivalent, and (vii) gel dosimeter to study the proton interaction, dosimetry and production of positron emitting radioisotopes. In addition, the present GUI program was coupled with a dedicated OpenGL 3D plotter program. The users could visualize the modeled geometry and proton range within the modeled phantom. The present GUI would reduce the possibility of errors and the time needed in preparing input scripts for proton irradiation and proton induced radioisotopes. In future works, we aim to further develop the present GUI and the OpenGL 3D plotter program by considering various types of ionizing radiations, geometries and materials for the PHITS MC package.

## Data Availability

All data files are available on Figshare: https://figshare.com/articles/software/Development_of_PHITS_graphical_user_interface_for_simulation_of_positron_emitting_radioisotopes_production_in_common_biological_materials/17054864
